# Indigenous Land and Sea Management Programs (ILSMPs) Enhance the Wellbeing of Indigenous Australians

**DOI:** 10.3390/ijerph17010125

**Published:** 2019-12-23

**Authors:** Silva Larson, Natalie Stoeckl, Diane Jarvis, Jane Addison, Daniel Grainger, Felecia Watkin Lui, Walalakoo Aboriginal Corporation, Bunuba Dawangarri Aboriginal Corporation RNTBC, Ewamian Aboriginal Corporation RNTBC, Yanunijarra Aboriginal Corporation RNTBC

**Affiliations:** 1 College of Business, Law and Governance, James Cook University and the Cairns Institute, Townsville, QLD 4811, Australia; 2 College of Business and Economics, University of Tasmania, Hobart, TAS 7005, Australia; 3 College of Business, Law and Governance, James Cook University and CSIRO Land and Water, Townsville, QLD 4811, Australia; diane.jarvis1@jcu.edu.au (D.J.); jane.addison@jcu.edu.au (J.A.); 4 College of Business, Law and Governance and the Indigenous Education and Research Centre, James Cook University, Townsville, QLD 4811, Australia; daniel.a.grainger@gmail.com; 5 Indigenous Education and Research Centre, James Cook University and the Cairns Institute, Cairns, QLD 4870, Australia; felecia.watkin@jcu.edu.au; 6 PO Box 1115, Derby, WA 6728, Australia; 7 PO Box 264, Fitzroy Crossing, WA 6765, Australia; 8 Hort St, Mareeba, QLD 4880, Australia; 9 Great Northern Highway, St George Ranges, WA 6728, Australia

**Keywords:** Indigenous land and sea management programs, wellbeing, impact evaluation, environment, country

## Abstract

Conservation and environmental management have been reported as offering opportunities to substantially improve the wellbeing of Indigenous people. Using the holistic wellbeing impact evaluation (W-IE) approach—well suited for use in Indigenous communities—we interviewed 190 Indigenous Australians across four communities. All communities were involved in the Indigenous land and sea management programs (ILSMPs). Our study explored the conceptualisation of ‘wellbeing’ by participants. In particular, we were interested in the aspects of wellbeing perceived to be affected by ILSMPs. Out of the 26 wellbeing factors explored, ‘Health centres’; ‘Language’; ‘Schools’; and ‘Safe community’ emerged as being of highest importance to the largest percentage of the respondents. When grouped using principle components analysis (PCA), the ‘Community and society’ domain emerged as the most important; accounting for 52% of the overall importance of all wellbeing factors. The second most important domain was the ‘Country and culture’, contributing 31%. Lastly, ‘Economic aspects’ contributed only 17%. Respondents believed that ILSMPs have played a considerable causal role in improving wellbeing, by positively changing factors most important to them. Specifically, 73% of perceived causal links were related to improvements in the ‘Country and Culture’ and 23% to ‘Community and Society’ domain. We thus conclude that land management for Indigenous people is much more than ecological or environmental management with ILSMPs, perceived to cause a wide range of cultural and social benefits. We also propose ways in which the future design of such programs could be improved to further increase benefits.

## 1. Introduction

Australian Aboriginal and Torres-Strait Islanders (hereafter referred to as Indigenous people) have managed their country for tens of thousands of years, undertaking a variety of different traditional land management practices. These practices involve much more than just managing the physical environment; Indigenous people also seek to manage the values, resources, stories and cultural obligations associated with a geographical area [1,2,3].

The Australian Federal Government has recognised the ecological value of Indigenous land management and has encouraged it through a range of funding programs, including inter alia, Indigenous Land and Sea Management Programs (ILSMPs); see [4] for a related typology of such programs. These programs have aimed to advance biodiversity conservation and support natural resource and heritage protection as well as create sustainable employment and economic opportunities for Indigenous people [5]. For example, in 2018, there were more than 800 Indigenous rangers employed across 118 groups [6] undertaking a variety of land and sea management activities, including the promotion of environmental and cultural objectives, community and stakeholder engagement, information management and knowledge exchange.

Although investment in ILSMPs was initially designed to support improved conservation and environmental management by the increased involvement of Indigenous people, over time, it has been more frequently reported as being able to improve the wellbeing of Indigenous people [7], with growing evidence that ILSMPs generate co-benefits. ‘‘Co-benefits’’ are a diverse range of benefits that reach far and above those associated with the environment (e.g., health, pride, confidence, and capacity building), and can accrue to a wide and diverse range of stakeholders, including Indigenous people [3,8] and funding bodies [5]. However, impacts are not necessarily all positive. Several researchers have noted that impacts are diverse—some being positive, and some negative (e.g., [9,10]) and some researchers have identified significant negative impacts (e.g., [11,12,13,14]). One such example of negative impacts is when a local group’s right to manage natural resources in the way that they see fit is constrained by a dependence upon external social actors or resources [15].

Proper assessment of programs is thus a non-trivial task—and reducing uncertainty and complexity in the identification, evaluation, and monitoring of co-benefits is emerging as a research priority [16]. Overwhelmingly, assessments focus on financial impacts (which may typically not be of intrinsic importance to Indigenous people, [17] and on impacts which are perceived as important by non-Indigenous people (i.e., government targets in ‘closing the gap’ [5]). However, relatively little is known about what ILSMPs and similar natural resources management programs mean to Indigenous people from their own point of view: value systems differ across cultures and what Indigenous people value most might differ from what a government program might consider of highest importance [15]. The broader literature suggests that in contrast to Western cultures, non-Western cultures’ conceptualisations of human wellbeing tend to be focused on the people–environment relationship and are therefore inextricably dependent upon the health and vitality of natural resources [2,18,19]. In non-Western cultures, natural resources are not only important for individual wellbeing—both physical and mental/spiritual—but are a base for perpetuating cultural traditions and communal identity [1,20,21,22,23]. 

In this study, we used the recently reported wellbeing impact evaluation (W-IE) approach [8] and interviewed 190 Indigenous Australians in four ILSMPs-engaged communities. We first set out to identify which aspects of wellbeing were perceived as important to interviewees (hereafter referred to as *important factors*) and to then learn more about their perceptions of the way in which ILSMPs had (or had not) impacted those important factors. We analysed data using Principal Components Analysis (PCA) to elicit key components of the conceptualisation of subjective ‘wellbeing’ and of the wellbeing impact of ILSMPs.

## 2. Materials and Methods 

### 2.1. Case Study Areas

**Figure 1:**
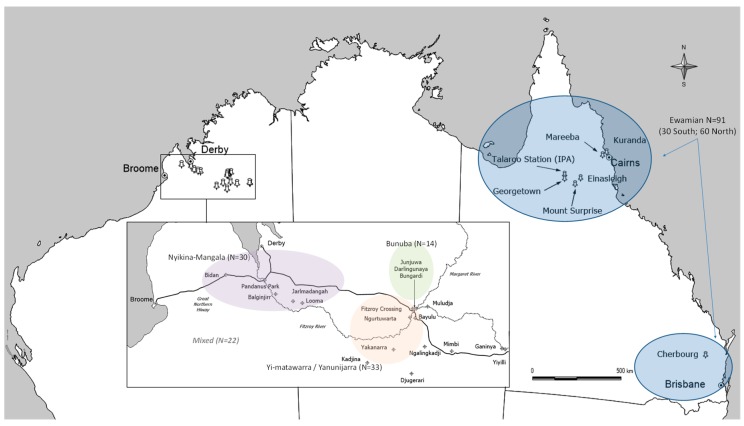
Communities in which people from partnering language groups predominately live.

The four ILSMP-active groups collaborating on this study comprised the Ewamian people from Einasleigh Uplands in north Queensland; and three other groups in the Fitzroy River Valley in Western Australia: Nyikina Mangala (working with the Walalakoo Aboriginal Corporation); Bunuba people (working with the Bunuba Dawangarri Aboriginal Corporation); and Walmajarri people (working with the Yanunijarra Aboriginal Corporation) (Figure 1).

The Ewamian people were dispossessed of their lands during the late nineteenth century with a significant proportion of people forcibly moved to areas including Cherbourg in south Queensland, Palm Island and Mona Mona missions in North Queensland. Although many remained in the general area, some living at the Georgetown Reserve and many employed as stockmen and domestic help through to the 1980s, nowadays there are Ewamian people living throughout Australia with significant populations in Brisbane/Cherbourg areas and other regional towns in North Queensland, such as Cairns, Mareeba and Kuranda (Figure 1). Very few Ewamian people currently live on their traditional country.

The three collaborating groups in Western Australia mainly live in the Fitzroy River valley, with significant populations in regional towns of Fitzroy Crossing, Derby and Broome (Figure 1). Those groups were largely dispossessed in the late 19th and 20th century, and subsequently, resided and worked on missions and local cattle stations. Many of these missions and cattle stations were on traditional country. Thus, although these people were, like the Ewamian people, dispossessed of their lands and rights through colonization, many still live on or relatively close to their traditional lands.

During the 1990s, all of the groups started organising into Aboriginal Corporations supporting applications for Native Title and land management. This process led to applications for Indigenous Protected Areas (IPAs) and/or ILSMPs. As a result, all four participating groups have recently or are currently engaged with various ILSMPs.

### 2.2. W-IE Method and Data Collection

The W-IE method for evaluating the wellbeing impacts of activities and programs [8], such as those related to improving environmental conditions, was used as the main data collection and analysis method in this study. Working alongside our Indigenous partners, we had a ***“yarn-up”*** (a term preferred to ‘interview’) with 91 Ewamian people in Queensland and 99 people from the Bunuba, Nykina-Mangala and Walmajarri people in WA. The ages of respondents ranged from 18 to 80 years, with a median of 44 years. A total of 176 respondents (88% of the sample) were 23 years of age or older at the time of the interview, hence, 18 years or older at the starting reference time (‘before the ILSMPs began’). Overall, 42% of respondents were male. English was reported as a language spoken at home by 52%, while others mainly spoke a mix of English, Kriol and traditional languages. Overall, 34% of respondents lived ‘on country’ (traditional lands), but this figure differs markedly between Western Australia (where most respondents live ‘on country’) and Queensland (only two Ewamian respondents lived ‘on country’). Only 30% of respondents came from households where formal (western) employment was the main source of household income. This study was approved by the James Cook University Human Ethics Committee, approval number H6500.

**Figure 2:**
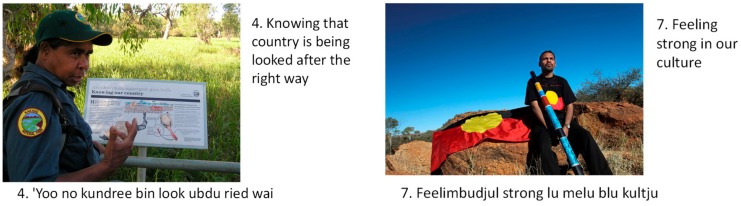
Examples of the wellbeing cards used in our primary data collection.

The first step in the W-IE method is the development of a context-specific and agreed-upon list of wellbeing factors of potential interest. The literature on wellbeing was reviewed to come up with an initial list of 45 factors, found to contribute to indigenous wellbeing in different contexts [8]. Factors were roughly categorised across different domains (health, social, environmental, economic, institutional), and then sent to partner organisations to screen for relevance. This narrowed the list down to 26. For each wellbeing factor that was selected in this way, we used words to describe it (English for Ewamian; English and Fitzroy Kriol for the Kimberley) and a photo to illustrate it, thus producing 26 ‘cards’ representing these different wellbeing factors. These descriptions were also discussed and agreed upon with representatives from partner organisations. An example of two such cards is presented in Figure 2; the final list of factors used in our study, with the wording used on cards and the abbreviations used in this paper, is presented in Table 1.

**Figure 3:**
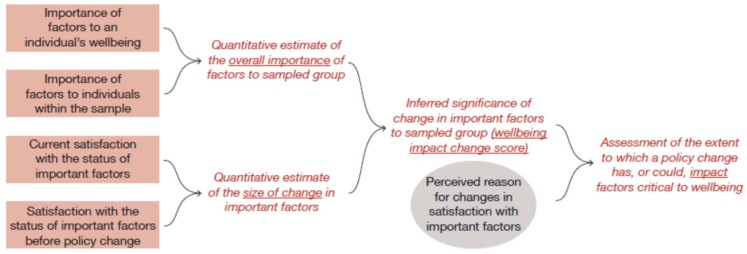
Conceptual framework for wellbeing-based method for impact evaluation (W-IE) used for data collection and analysis in this study. Information elicited directly from intended program beneficiaries is shown in boxes (quantitative data) and ellipse (qualitative data), information inferred from responses to direct questions is shown in italics (without frame).

Following the W-IE conceptual framework (Figure 3), we worked with our partners to have a ‘yarn-up’ with people, analysing responses in related steps:

1. We asked individuals to review each of the 26 (wellbeing) factor cards and sort them into two separate groups—those that were not important to their own wellbeing and those that were. Having set aside the unimportant factors, we asked people to select the six factors that were deemed to be the most important to their own personal wellbeing. For each individual, we thus had three groups of factors: those deemed unimportant, a ‘middle’ group, and the most important.
2. We asked individuals to focus on the six factors that were selected as most important to their wellbeing and to rate the importance of those core factors on the scale from 1 to 10 (see boxes in Figure 3). Hence, we obtained information on both how many individuals within our sample selected a factor as being of most importance, and (for selected factors) how important that factors is. This allowed us to estimate the ‘*Overall Importance*’ of factor(s), as the % of respondents selecting a factor multiplied by mean importance assigned by those who selected (and thus rated) the factor.
3. We also asked individuals to tell us how satisfied they were with each core factor, both ‘now’ (at the time of the ‘yarn-up’) and 5 years previously (before the Indigenous Land and Sea management occurred) (see boxes in Figure 3). Subtracting one satisfaction score from the other allowed us to generate a quantitative measure of ‘*Size of change*’.
4. We multiplied estimates of the change in satisfaction (step 3) by estimates of the overall importance scores (step 2) to estimate the significance of perceived change to wellbeing (*Wellbeing impact change score*).
5. Whenever a change was noted, respondents were asked what had happened to cause the change. We were careful not to ‘lead the witness’—and did not explicitly ask if they perceived a link to ILSMPs. Responses to this open-ended question were then coded, explicitly noting whether the respondent had attributed changes to, or associated changes with, native title or ILSMPs (see ellipse in Figure 3)—termed: *‘Link to ILSMPs’*. The ‘strength’ of the link between any individual factor and ILSMPs was measured by calculating the percentages of respondents who had selected a factor as important, who also linked observed changes in satisfaction with that factor to an ILSMP. Combining qualitative responses with quantitative scores (step 4) thus provides inferential information about the extent to which a program (in this case ILSMPs) is seen as having impacted ‘important factors’—termed: program’s *Impact.*

We compared two cohorts (Queensland and Western Australia) for potential differences in importance and satisfaction scores (Supplementary Materials). There were no statistically significant differences in life satisfaction (LS) now nor in changes in LS overall between the two cohorts; however, respondents in Western Australia assigned statistically significantly higher scores to the importance of Legal protection and Strong in culture (at 95% CI) and to Language, Sharing knowledge and Community spirit (at 99% CI). We also compared responses of people who reported living on land, versus those who did not (Supplementary Materials). Legal protection, Strong in Culture, Language and Community Spirit were more important to those living on the Country than others (at 95% CI but not at 99%). Again, there were no statistically significant differences in life satisfaction (LS) now nor in changes in LS overall between the two cohorts, although some differences in satisfaction with individual wellbeing factors were noted. We propose that differences found between WA and QLD cohorts are potentially due to large percentage of people in WA living on country (70% of all WA respondents; compared to only 2% for QLD). Taking this analysis forward to the comparison of perceptions of links between ILSMPs and wellbeing factors, we found that both cohorts have very comparable results, but that Language and Sharing knowledge recorded somewhat stronger links to ILSMPs in QLD than in WA. This might be for the reason that unfortunately Ewamian Language did not survive and is no longer spoken; however, Ewamian people are working with linguist specialist on a program to ‘rebirth’ the language.

Future research could very usefully examine the links between selected factors, perceptions of ILSMP ‘impact’ and various socio-demographics (including, but not limited to location, age, income, gender). One would expect that different factors would be important to the wellbeing of different people. Noting the important additional insights that such individual-level analysis could add, we focus here on perceptions and impacts at a whole-of-community level, hence treating the two cohorts as one sample for further analysis.

### 2.3. Conceptualising Wellbeing and the Impact that ILSMPs Have on Overall Wellbeing

Many wellbeing factors are conceptually similar and/or inherently related. We thus sought to better understand the relationship between wellbeing factors, their collective contribution to overall wellbeing, and the impact that ILSMPs have on wellbeing overall.

First, we set out to identify (statistically) separable groupings within our 26 factors, based on the importance assigned to each. The level of importance (least, middle, most) assigned to each factor by each individual respondent were coded such that the factors rated least important were assigned a score of 0, middle importance a score of 1 and most important a score of 2. We then combined these scores for the entire sample of 190 respondents (The first 67 individuals interviewed, all from Ewamian, were not asked about Bush tucker, thus were only asked about 25 factors rather than 26. For the PCA, we allocated a middle-importance ranking to this factor for all of these respondents; that being the most frequent response given by those who were asked about this factor), and examined whether the factors could be grouped into a smaller number of components reflecting different domains of life. We used principle component analysis (PCA), with Varimax rotation and Kaiser normalization, within the IBM SPSS statistical package (version 25) to do this. We also repeated the PCA analysis using alternate rotation methods (Oblimin, Promax, Quartimax and Equamax rotation methods); the results from different approaches provided virtually identical results, thus providing multiple lines of evidence from which we are able to draw what we feel to be reasonably robust conclusions (results from alternate analysis available from the authors on request). From examining the scree plot, we found that these 26 factors collapsed into three separable components, which we refer to here as representing different domains of wellbeing. We also tested the robustness of our findings by testing the impact of separating the factors into 4 components, again finding our results to be highly robust. For each domain identified by the PCA, we

(1) Added all importance scores for factors within that domain, and then divided through by the sum of all overall importance scores. This provides information about the relative importance of each domain to overall wellbeing (*Domain Importance*); and
(2) Added the number of times that responses to our open-ended questions about the perceived causes of observed change to each included factor was linked to ILSMPs, and then divided through by the sum of all ILSMP links. This provides information about the relative ‘impact’ of ILSMPs on each domain (*ILSMP impact on Domain*).

## 3. Results

### 3.1. Wellbeing Impact Evaluation (W-IE)

The wellbeing factors in Table 2 are ordered by their ‘*Wellbeing Impact Change Score*’ (calculated in line with the W-IE step 4 as described in methods and reported in the last column of the Table). This score takes into account both the ‘Overall importance’ of factors to wellbeing, and the reported ‘Change in satisfaction’ with that factor over the last five years. The satisfaction change might be a negative or a positive change, so the ‘Wellbeing Impact Change Score’ might also be negative or positive. ‘Country looked after’, ‘Schools’, ‘Legal right to country’, ‘ICT’ and ‘Role model’ emerged at the top of the table, indicating these factors are of high importance to many people, who are more satisfied with their condition now than they were five years ago.

The first column of Table 2 reports the *Overall Importance* of factors (W-IE step 2). All factors recorded mean importance scores over 9 (out of 10), so the difference in the Overall Importance score is really based on the percentage of respondents selecting a factor (i.e., the lower the score, the lower the number of people selecting it as highly important). It can be observed that ‘Health centres’; ‘Language’; ‘Schools’; and ‘Safe community’, received the highest scores, indicating that these factors are of high importance to the largest percentage of the respondents.

The *Size of the Change* in satisfaction with each factor (calculated as satisfaction now—satisfaction before) is also presented in Table 2. The highest positive change in satisfaction (more than 2 points of change) was recorded with the factor ‘ICT’, and this change was attributed to installation of new mobile telephone towers and hence better coverage on country. Respondents also reported large positive changes (more than 1.5-point change) in satisfaction with the wellbeing factors: ‘Country looked after’; ‘Legal protection’; ‘Legal right to country’; ‘Power to influence’; and ‘Own business’. However, a number of factors, namely, ‘Law enforced’; ‘Local jobs’; ‘Bush tucker’; and ‘Social ills’, were reported as deteriorating over time, that is, satisfaction of respondents with the situation in relation to factors was lower now than it was 5 years ago.

In the next step we explored perceived reasons for changes in satisfaction. In an effort to minimize problems of social desirability or strategic bias, we did not in any way prompt respondents about ILSMPs: they were simply asked, in their perception, what created change. We do, however, acknowledge that respondents were aware that the study related to ILSMPs, so cannot guarantee absence of such biases. Nevertheless, the results suggest that responses were not all strategic: several factors were not linked to ILSMPs by any of the respondents, rather, they reported other developments in their personal lives, community, or society at large. For example, satisfaction with the ‘Schools’—a very important wellbeing factor to many respondents (overall importance score of 310)—recorded a positive 1.33-points change; but this change was attributed to better facilities and not linked to ILSMPs by any of the respondents. Similarly, respondents reported significant increases in satisfaction with ICT—linking that to improvements in mobile communications, without any mention of ILSMPs.

**Figure 4:**
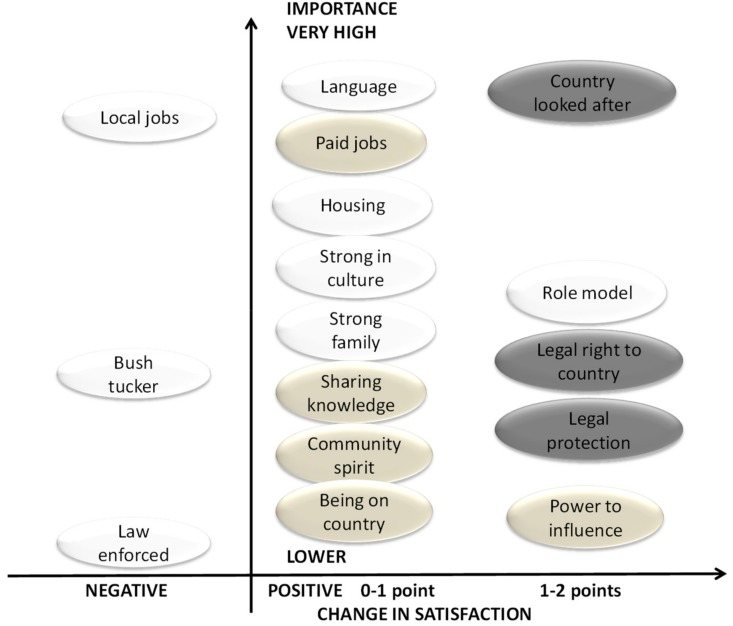
Visual mapping of wellbeing factors perceived by respondents as linked to ILSMPs, in relation to their importance (vertical axes) and change in satisfaction (horizontal axis). Stronger link to ILSMPs is indicated by darker shade.

However, respondents linked improvements in satisfaction with several other ‘most important’ factors to ILSMPs. These factors included: ‘Language’; ‘Local jobs’; ‘Country looked after’; ‘Paid jobs’; ‘Housing’; ‘Strong in culture’; ‘Strong family’; ‘Role model’; and ‘Legal right to country’. The factors most ‘strongly’ linked to ILSMPs were ‘Country looked after’; ‘Legal protection’; and ‘Legal right to country’. Figure 4 presents visual mapping of wellbeing factors perceived by respondents as linked to ILSMPs (darker shading identifies stronger links). Factors are placed in a matrix where the vertical axis represents importance; and the horizontal axis represents change in satisfaction. It can be noted that factors with the strongest reported links to ILSMPs are the ones receiving high positive satisfaction change score.

Satisfaction was reported as decreasing for three factors linked to ILSMPs: ‘Law enforced’; ‘Bush tucker’; and ‘Local jobs’ (Figure 4). Although the overall satisfaction with these factors had fallen during the previous 5 years, qualitative responses to questions about reasons for change all indicated that the perceived impact of ILSMPs on these factors was positive. For example, in terms of access to bush tucker, one respondent stated, “*Connecting to country, fishing and hunting, is a healing process*” (RWA13); while ranger programs supported by ILSMPs were seen as positively impacting on local jobs (and with the potential to do more so in the future): “*For wellbeing of people and community, so people don’t have to go looking for jobs in other places, and money they get paid goes back into community*” (RWA73).

### 3.2. Conceptualisation of Wellbeing Domains and the Impact that ILSMPs Have on those Domains

The PCA components, and the factors which were grouped into each component resulting from the PCA, along with the component loading scores, are set out in Table 3. PCA calculates a loading score (ranging from 0 to 1) for each of our 26 factors for each component, with the loading score indicating how strongly the factor contributes to each principle component identified. We classified each factor into the component where the factor received the highest loading score. Graphical analysis of the PCA results are set out in the Supplementary Materials. We refer to each of the principle components, each containing a separate group of factors, as a ‘domain’.

Interestingly, the domains are drawn on distinctly cultural lines. The large and positive factors within the first domain all reflect strongly Indigenous cultural factors. The only factor included in this domain that has a negative link is the one that is associated with ‘having good quality clinics nearby’, indicating that western health facilities might not be seen as complementary to Indigenous cultural factors. Domains 2 and 3 reflect more Western goals and concepts; differentiated according to whether the factors largely describe benefits that accrue to the community or society more generally (domain 2), and those where the benefits are of a more individual and more economic nature (domain 3). We developed descriptive domain names for each component identified by the PCA, based on the combination of factors most heavily loaded into each component. It is also notable that should the PCA analysis be used to extract four groupings rather than three, the first grouping, Country and Culture is completely unchanged, indicating the robustness of the segregation between domains along distinctly cultural lines.

The top row of Table 4 shows *Domain importance*, clearly highlighting that factors associated with *Community and Society* have received most of the importance scores (52%). Each column of Table 4 lists the factors which loaded within that domain (Table 3), and counts the number of times that responses to the open ended question about the perceived causes of change mentioned ILSMPs (‘No. links’). The bottom row counts those mentions—reporting also the percentage of ILSMP ‘mentions’ that are associated with each domain. It can be noted that 73% of all links made between ILSMPs and wellbeing are for factors associated with *Country and culture*. Factors associated with *Community and society* accounted for 23% of all links, while factors associated with the *Individual and economy* were linked to ILSMPs in 4% of cases.

## 4. Discussion

Environmental, social and economic benefits of land management programs are many and accrue to many [5,16,24], but in this paper, we concentrate on benefits to Indigenous wellbeing. Different cultures conceptualise things differently [25]; so it is important that Indigenous peoples have the opportunity to conceptualise their own wellbeing. By designing a method that prompts respondents themselves to create their own ‘wellbeing functions’ (sets of wellbeing factors that they perceived as the most important to them) we circumvent the issue of delimitation, that is, which types of changes are valued as important and by whom [8].

The literature suggests that many Indigenous peoples view themselves as ‘a part of’ (rather than ‘apart from’) the natural world and reveal powerful ecological frameworks or ways of ‘knowing and doing’ [25]. Furthermore, human and nonhuman worlds exchange material, energy, and spirits, and the past and future characterise the present [26]. Land, or ‘Country’, is central to the formation of identity in Australian Aboriginal people, where country is not seen as something separate from the self [27]. Rather, Country forms one aspect of the self and the identity of the individual and the group. Australian Aboriginal people perceive ‘Country’ as being alive and in this sense, capable of thought and reflection [27]. Country provides a sense of wellness, that is, the wellness of the people reflects the wellness of Country. Links to Country are not only important for individual wellbeing, but are a base for perpetuating cultural traditions and communal identity [1,20]. This holistic approach can be found in the definition of Aboriginal health asserted by the Australian National Aboriginal and Community Controlled Health Organisation (NACCHO), the peak body for Aboriginal community controlled health services in Australia: “Aboriginal health” means not just the physical wellbeing of an individual but refers to the social, emotional and cultural wellbeing of the whole Community in which each individual is able to achieve their full potential as a human being thereby bringing about the total wellbeing of their Community.” [28]. These interrelated characteristics underpin notions of health and wellbeing for Indigenous communities, hence, land management is for Indigenous peoples much more than just managing the physical environment [1,2,3]. Health approaches that neglect to consider the relationship between health, Country and community do little to close the (health) gap between Indigenous and Non-Indigenous Australians.

Colonial displacement from traditional lands (‘country’) resulted in a loss of traditions and traditional culture, and is contributing to social and health problems of Indigenous Australians [29,30]. The disruption to well established patterns of living, dispossession of land, marginalisation through various government acts and discrimination, has led to trauma (see for example [27,31,32]. Conversely, re-establishing links to country, through both hands-on environmental works and contributions to its management (power over what works are done and how and how are these works selected and implemented) is seen as a potential opportunity for improving Indigenous wellbeing [15,33]. In our study, we find that wellbeing factors related to ‘Country and culture’, which received 31% of all scores for wellbeing importance, were perceived as strongly linked to ILSMPs and related processes, suggesting that changes due to such programs are valued as highly important by the participants themselves. Of particular significance is access to country, both in terms of the legal right to access and the ability to access (resources), as this is a catalyst for what activities can be carried out on country. Thus, factors such as being on country; strong in culture; sharing knowledge, are all perceived as related to access. Further, this connection may not necessarily be satisfied by simply living on country. Instead, connection to country is affected by the potential for autonomy, access and use of country as desired [34]. The need for autonomy or control has important implications in the context of creating opportunities for development [15].

The only factor included in ‘Country and culture’ domain that has a negative link is the one that is associated with ‘having good quality clinics nearby’ (‘Health clinics’). Health clinics received the highest overall importance score of all wellbeing factors explored (362 points) but the lowest satisfaction change score (0.23 out of 10). Conceptually, ‘access to health services’ can refer to whether health services are geographically accessible, financially accessible, culturally accessible, or whether they have the workforce or capacity to see patients when they need assistance. Although the bulk of the medical costs in Australia are covered by government through Medicare and Health Care card initiatives, financial implications of remaining ‘out-of-pocket’ payments on low income families are worth considering. Recent reports in Australia suggest that the geographical and cultural accessibility remain an issue for Indigenous population [35,36]. As per Australian Standard Geographical Classification System, our case study regions are located in ‘very remote’ parts of Australia, where the proportion of people who are Indigenous is high (in 2011, 45% of people living in very remote areas were Indigenous; compared to 3% of the total Australian population [35]). The coverage with the primary health care services in these areas is improving, however, specialist care services remain sparse. For example, less than 20% of Indigenous women in very remote areas live within a one-hour drive from a hospital with a public birthing unit [36]. Cultural accessibility also remains an issue, as the current literature suggests that western medical facilities may be seen as crowding out important Indigenous cultural practices, which provide more holistic approaches to healing. Several studies have identified cultural barriers to Aboriginal people accessing ‘western’ medical services, including misunderstanding, fear of death, fatalisation, shame and preference for traditional healing (see for example [37,38,39,40]. For Aboriginal patients, the focus on interpersonal relationships between themselves and health practitioners is paramount [39], underscoring the need to build staff cultural competency and enhance cultural safety of western medical facilities. The negative link score between health clinics and Indigenous culture is, however, interesting in that it suggests they might not be seen as complementary. Further research could usefully shed light on this finding.

Some of the wellbeing factors in the ‘Community and society’ domain were also perceived as linked to the ILSMPs, such as ‘Community spirit’ and ‘Power to influence’. The latter is in line with calls by Indigenous people for a new policy approach that incorporates Indigenous knowledge and values, and the meaningful participation of Indigenous peoples [41], and where development is conceptualised as ‘control, leadership, empowerment and independence’ in line with Sen’s development as freedom [15]. Interestingly, this domain presents a fair amount of overlap with the Australian Government ‘Closing the Gap’ initiative [42], which aims at closing the attainment gap between Indigenous and non-Indigenous Australians in life expectancy, child mortality, early childhood education, school attendance, literacy and numeracy, year 12 attainment and employment.

This study has policy implications in that it provides some insights in how the design of ILSMPs and similar programs can be improved to further increase perceptions of wellbeing. ILSMPs are already contributing to the ‘Country and culture’ wellbeing domain, but further impacts could be made on the factors in the ‘Community and society’ domain, which was identified by participants as the most important one. Specifically, programs could design activities to tackle social ills and support community spirit, and could play a greater role in promoting and supporting Indigenous role models among Indigenous rangers engaged in land management. An interesting area for creating change is ‘Bush tucker’ (traditional foods available in the environment), which in this study is linked not to ‘Country and culture’ but rather economic aspects. Further understanding of role of bush tucker in wellbeing conceptualisation, and investigations of its benefits, might be worth considering, including recovery of traditional plants and agriculture as a way of promoting wellbeing through cultural maintenance and traditional knowledge [43]. Future research could also benefit from examining the links at individual level, between individual socio-demographic characteristics of respondents such as location, age, income and/or gender; and wellbeing factors selected and perceived ILSMP ‘impact’.

## 5. Conclusions

Wellbeing of Indigenous people is a wide holistic concept, strongly linked to ‘Country’. Land management is thus much more than ecological or environmental management and Indigenous land and sea management programs (ILSMPs) do also create a wide range of cultural and social benefits. Based on our results, we can conclude that ILSMPs have played a significant role in improving the wellbeing of study participants by positively changing some of the things most important to them. We also show how the participatory wellbeing approach used here can inform improved design of land management and similar programs to further increase social and cultural co-benefits.

## Figures and Tables

**Table 1:** The 26 wellbeing factors explored in this study.

Wellbeing Factor	Referred to in the Paper as
Having enough power to influence decisions that affect my life (e.g., decisions about housing, how to spend money, etc.)	Power to influence
Being a role model or having role models in the community	Role model
Having the legal left to use/access country	Legal left to country
Knowing that country is being looked after the left way	Country looked after
Being out on country (for any reason)	Being on country
Obtaining legal protection for places, knowledge or practices with important cultural value	Legal protection
Feeling strong in our culture	Strong in culture
Making sure language is not ‘lost’ (spoken regularly and/or written down)	Language
Sharing knowledge (traditional and new) within and outside community	Sharing knowledge
Having houses that are in good condition and not overcrowded	Housing
Having good quality schools and training centers close by	Schools
Having good quality clinics and hospitals close by	Health centres
Reducing how much I use grog, smokes or gunja	Social ills
Feeling good and strong in my body and mind	Strong person
Knowing my family are feeling good and strong in their bodies and mind	Strong family
Knowing that people in our community feel good about each other and work together to help when needed	Community spirit
Knowing that my community is a safe place for me and my loved ones	Safe community
Knowing that people who behave outside the law (or Aboriginal law) are punished	Law enforced
Having a paid job	Paid job
Enjoying the work I do (paid or unpaid)	Work satisfaction
Having more money	More money
Having my own business	Own business
Being able to save money for big purchases (e.g., car or house)	More saving
Having jobs available in my local community	Local jobs
Being able to use a mobile phone and internet in our community and on country	ICT (Information and Communications Technology)
Consuming traditional foods	Bush tucker

**Table 2:** Ranking of the wellbeing factors based on their Wellbeing Impact Change Score, indicating reported overall importance of each factors and the perceived size of the change in satisfaction.

Wellbeing Factor	Overall Importance	Size of Change in Satisfaction	Wellbeing Impact Change Score
Country looked after	262	1.73	453
Schools	310	1.33	412
Legal right to country	235	1.69	397
ICT	137	2.67	366
Role model	252	1.35	340
Legal protection	184	1.55	285
Power to influence	158	1.79	283
Language	329	0.73	240
Social ills	101	−1.89	−191
Strong family	269	0.66	177
Paid job	274	0.59	161
Strong in culture	268	0.6	161
Strong person	225	0.63	142
Local jobs	288	−0.48	−138
Work satisfaction	122	1.02	124
Own business	68	1.69	115
Housing	280	0.35	98
Being on country	167	0.53	88
Health centres	362	0.23	83
Sharing knowledge	206	0.4	82
More saving	91	0.87	79
Safe community	303	0.23	69
Community spirit	204	0.32	65
Bush tucker ^1^	224	−0.26	−58
Law enforced	89	−0.51	−45
More money	82	0.41	33

^1^ Not asked to initial 67 Ewamian participants: thus asked to 24 Ewamian and all in WA (99 participants).

**Table 3:** Component loading scores from the Principle Components Analysis, classifying factors by the principle component into which the factor loads based upon the PCA loading scores.

Domain 1: Country and Culture	Domain 2: Community and Society	Domain 3: Individual and Economy
Being on country (0.692)	Paid job (0.627)	ICT (0.718)
Legal protection (0.659)	Safe community (0.578)	More money (0.654)
Strong in culture (0.566)	Strong person (0.505)	Law enforced (0.564)
Legal right to country (0.563)	Work satisfaction (0.482)	Housing (0.525)
Language (0.558)	Schools (0.477)	Bush Tucker (0.492)
Country looked after (0.549)	Strong family (0.394)	Own business (0.445)
Sharing knowledge (0.544)	Local jobs (0.382)	More savings (0.420)
Health centres (−0.397)	Community spirit (0.338)	
	Role model (0.295)	
	Social ills (0.288)	
	Power to influence (0.166)	

**Table 4:** Grouping of wellbeing factors as a result of PCA (Domains), with the reported links between ILSMPs and factors in each domain and the % of Overall Importance for each domain.

Domain 1: Country and Culture	No. Links	Domain 2: Community and Society	No. Links	Domain 3: Individual and Economy	No. Links
31% of Overall Importance		52% of Overall Importance		17% of Overall Importance	
Country looked after	19	Community spirit	6	Law enforced	1
Legal right to country	14	Paid job	4	Housing	1
Legal protection	11	Power to influence	4	Bush Tucker	1
Sharing knowledge	7	Local jobs	3	ICT	
Language	5	Role model	2	More money	
Strong in culture	5	Strong family	2	More savings	
Being on country	4	Strong person		Own business	
Health centres		Safe community			
		Work satisfaction			
		Schools			
		Social ills			
Total links	65	Total links	21	Total links	3
% of all links made	73	% of all links made	23	% of all links made	4

## Supplementary Materials

The following are available online at https://www.mdpi.com/1660-4601/17/1/125/s1.
