# Supplementary material for: Indigenous Land and Sea Management Programs (ILSMPs) Enhance the Wellbeing of Indigenous Australians
Source: Int J Environ Res Public Health. 2019 Dec 23;17(1):125. doi: 10.3390/ijerph17010125 (PMC6981778; doi:10.3390/ijerph17010125)
Supplement: Supplementary file 1 [file ijerph-17-00125-s001.pdf]

Supplementary Material – graphical output from principle components analysis

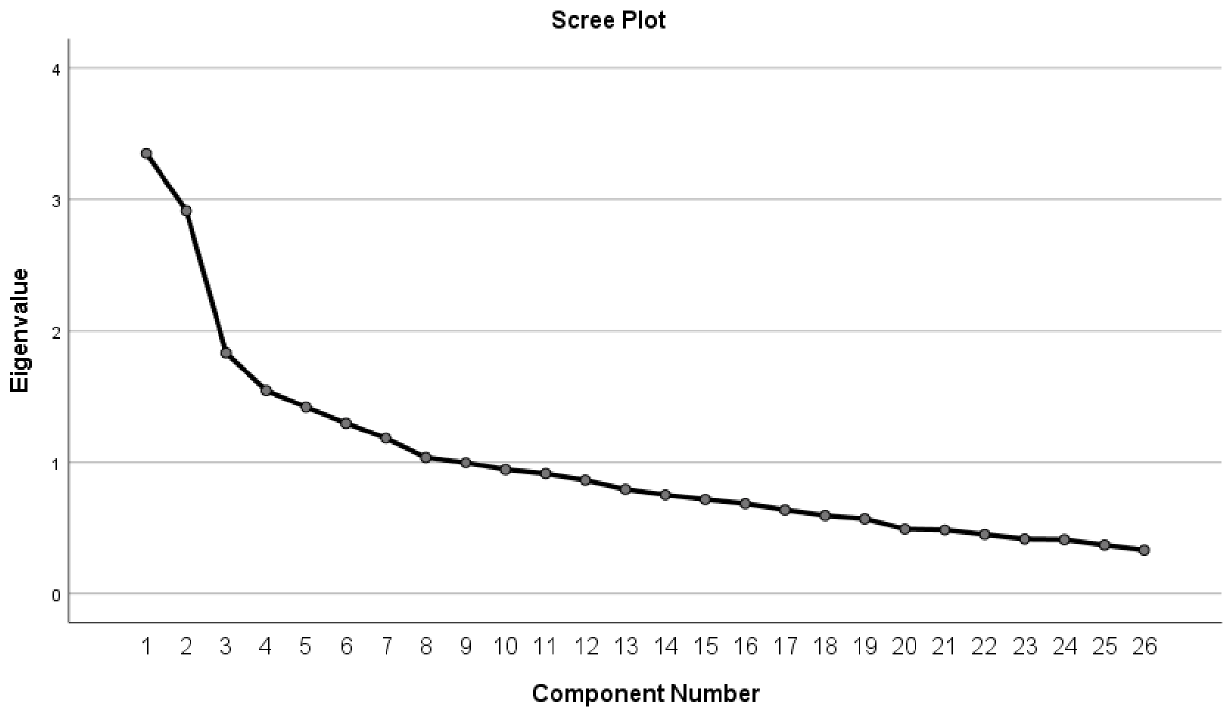

Figure S1. Scree plot indicating inflexion justifying retaining 3 factors.

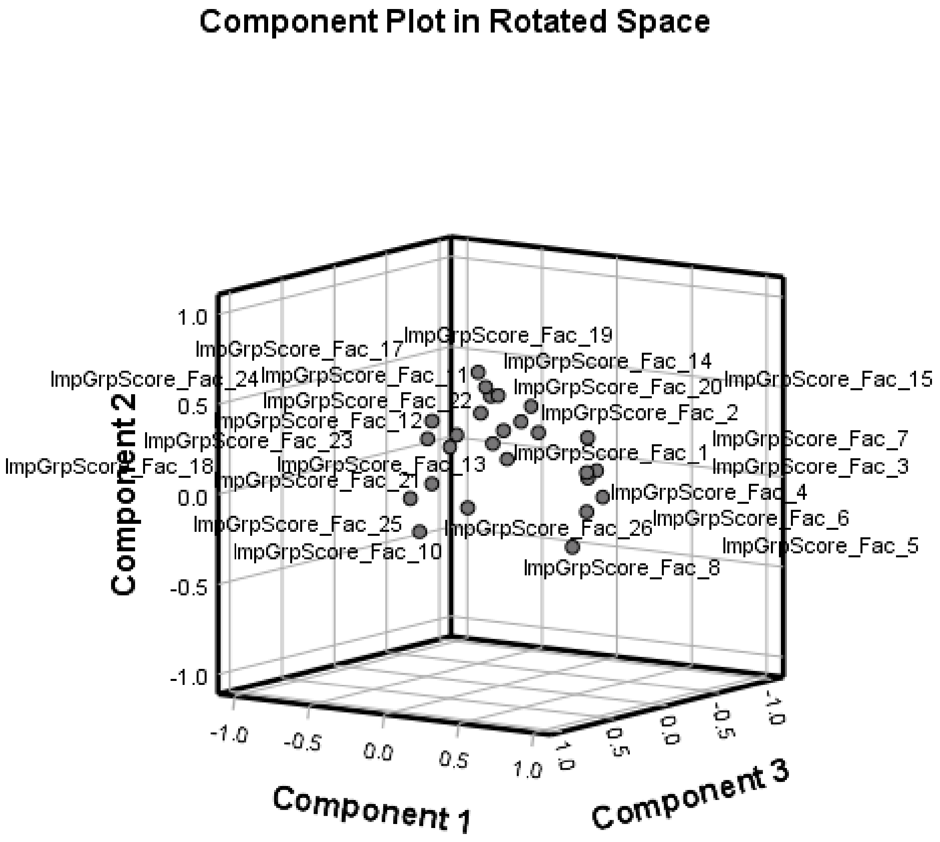

Figure S2. PCA graphical output from SPSS – Component plot in 3D rotated space showing all three components

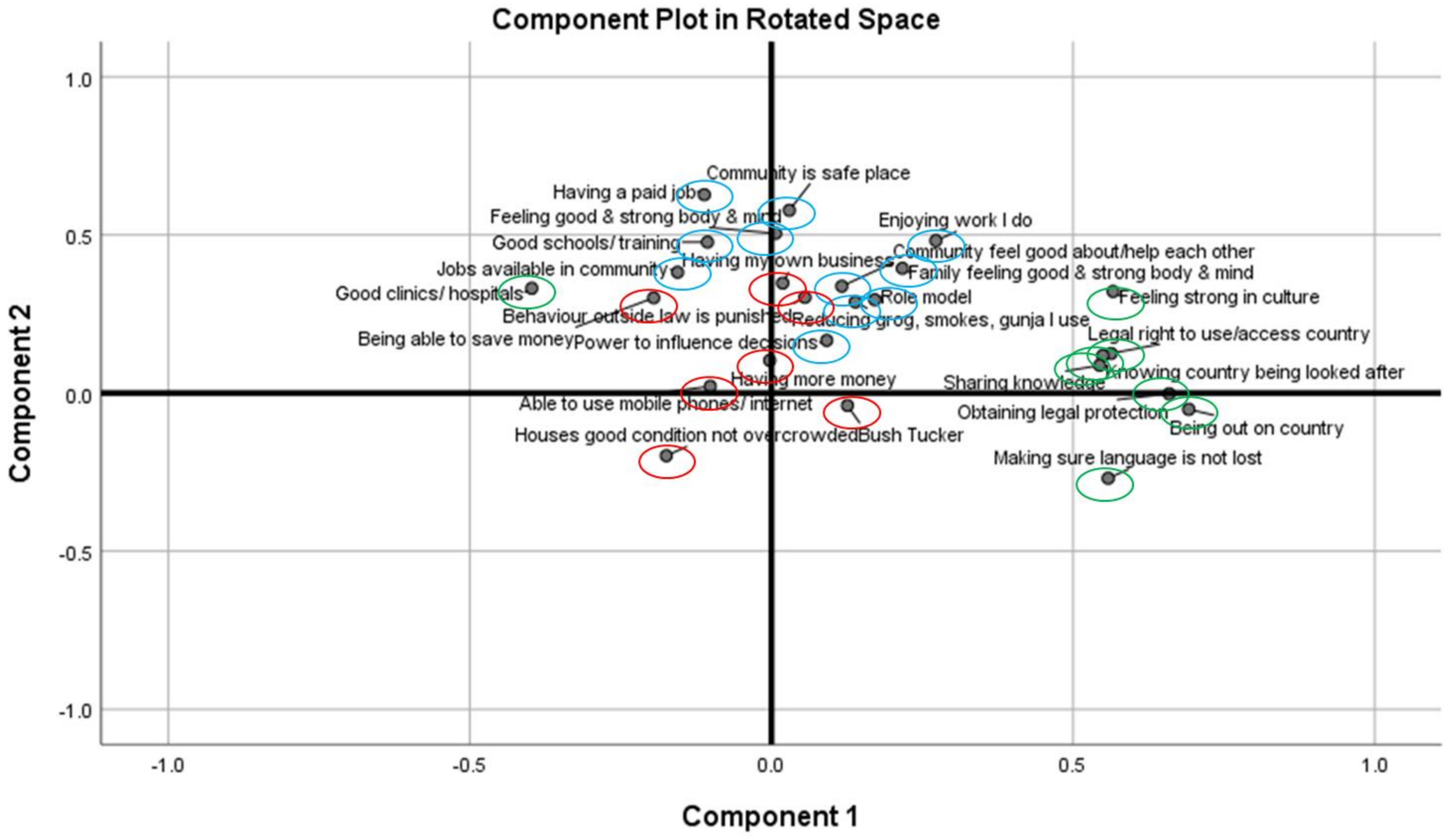

Figure S3. PCA output from SPSS – Component plot in rotated space showing component 1 (Domain 1: Country and Culture) and component 2 (Domain 2: Community and Society). Factors loading primarily to component 1 are circled in green, factors loading to component 2 are circled in blue and factors loading to component 3 are circled in red.

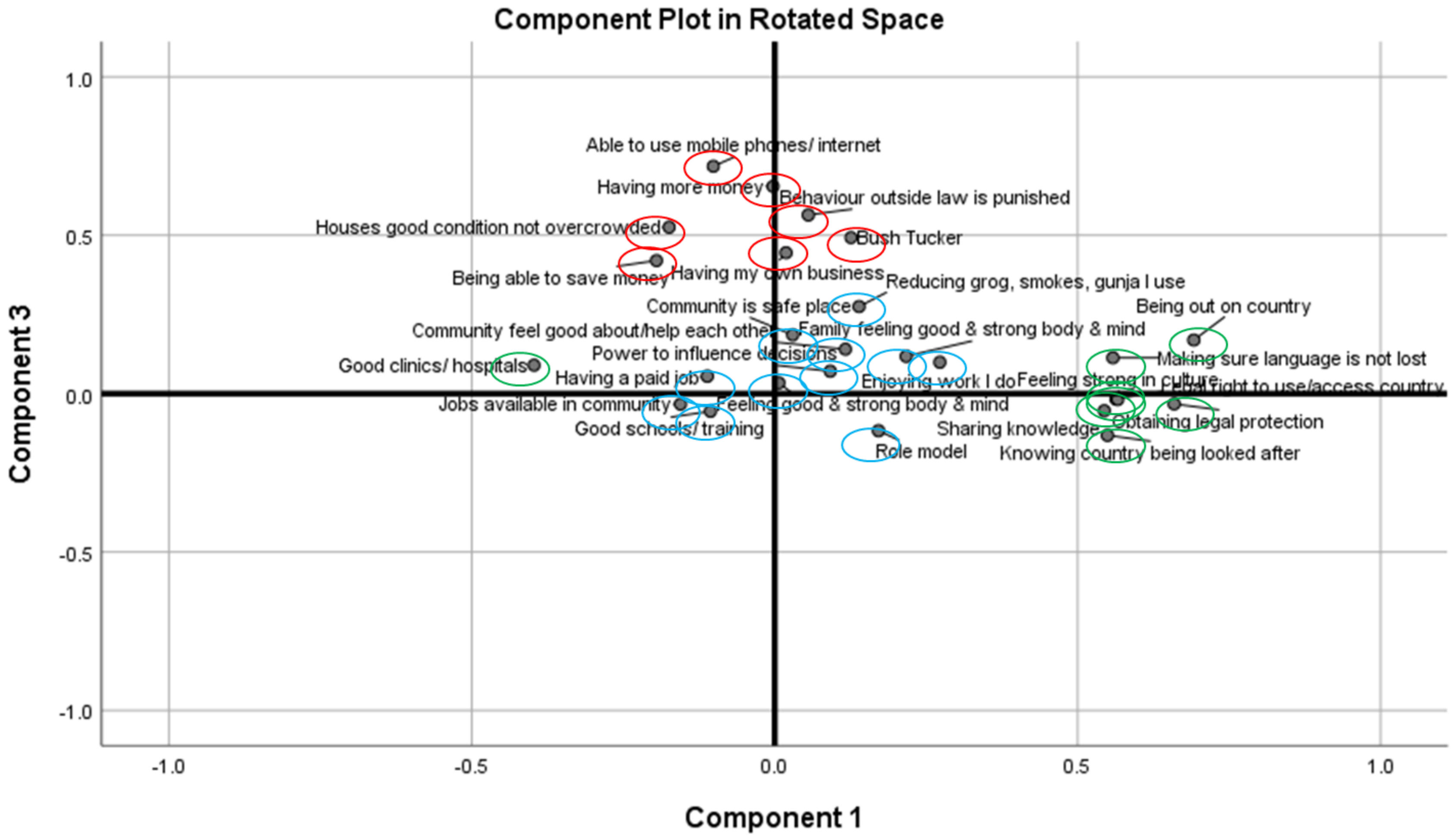

Figure S4. PCA output from SPSS – Component plot in rotated space showing component 1 (Domain 1: Country and Culture) and component 3 (Domain 3: Individual and Economy). Factors loading primarily to component 1 are circled in green, factors loading to component 2 are circled in blue and factors loading to component 3 are circled in red.

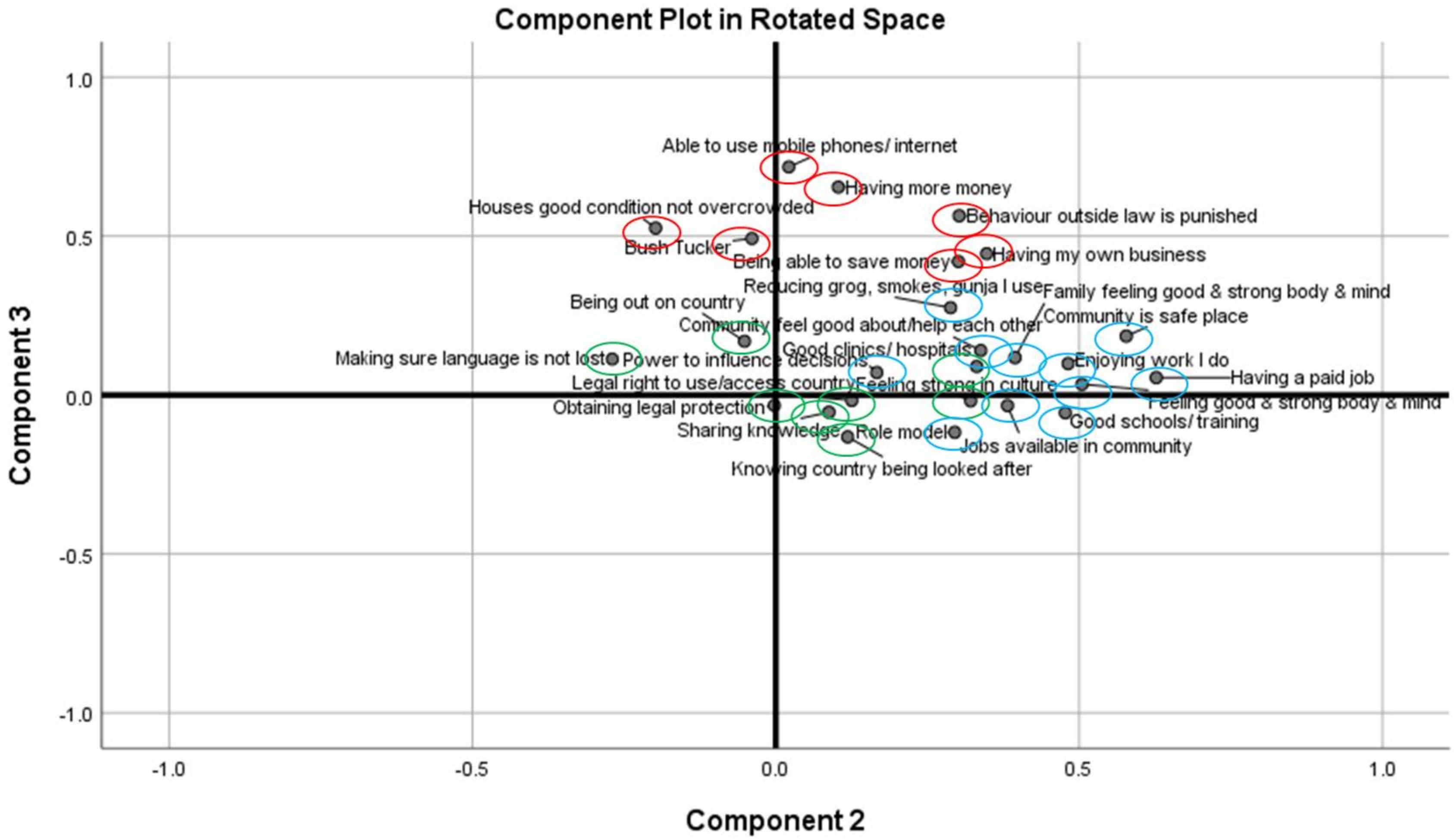

Figure S5. PCA output from SPSS – Component plot in rotated space showing component 2 (Domain 2: Community and Society) and component 3 (Domain 3: Individual and Economy). Factors loading primarily to component 1 are circled in green, factors loading to component 2 are circled in blue and factors loading to component 3 are circled in red.
